# World Beliefs Moderate the Effects of Trauma and Severe Illness on Emotional Distress

**DOI:** 10.1111/jopy.70031

**Published:** 2025-11-12

**Authors:** Nicholas Kerry, Janna Hämpke, Adrienne Wood, Shelly Tsang, Kyle Barrantine, Shigehiro Oishi, K. C. White, Jeremy D. W. Clifton

**Affiliations:** ^1^ University of Pennsylvania Philadelphia Pennsylvania USA; ^2^ University of Vienna Vienna Austria; ^3^ Ludwig Maximillian University of Munich Munich Germany; ^4^ University of Virginia Charlottesville Virginia USA; ^5^ University of Chicago Chicago Illinois USA

**Keywords:** anxiety, depression, negative life experiences, primal world beliefs, trauma

## Abstract

**Objective:**

Severe illness and trauma can cause significant psychological distress, but individuals differ in their responses. This research tested whether world beliefs—fundamental assumptions about the nature of the world—moderate the relationship between negative life experiences and emotional distress.

**Method:**

Study 1 compared individuals with chronic illnesses (cystic fibrosis or cancer) to healthy controls on measures of anxiety, depression, and world beliefs. Study 2 analyzed longitudinal data from university students assessed before and after a campus mass shooting, focusing on the Safe world belief as a moderator of stress.

**Results:**

In Study 1, people with chronic illness showed substantially higher anxiety and depression than controls at low levels of Improvable, Regenerative, and Just world beliefs, but did not differ at high levels of those beliefs. In Study 2, students low in Safe belief reported increased stress both shortly after and 4 months after the shooting, while those high in Safe belief showed no increases. Other positive world beliefs were less effective moderators.

**Conclusions:**

World beliefs appear to buffer individuals from emotional distress following severe illness or trauma. Further, the specific content of these beliefs, as well as their valence, appears important for emotional resilience.

Highly negative life experiences can have devastating impacts on psychological wellbeing (e.g., Spinhoven et al. 2010; Strauss et al. 2020; Yao et al. 2019). For instance, patients recently diagnosed with cancer report lower quality of life and life satisfaction, and are four to eight times more likely to be depressed or anxious (Haun et al. 2014; Williams et al. 2016). Similarly, traumatic experiences, such as childhood sexual abuse, are tied to substantially elevated anxiety, depression, and suicide risk (Lindert et al. 2014; Pegram and Abbey 2019; Zatti et al. 2017). However, not everyone who has a major negative life experience shows lasting increases in anxiety and depression (e.g., Bonanno et al. 2005; Deshields et al. 2006; Mancini et al. 2016). So, why are some people more likely to experience emotional distress than others? An important goal for researchers is to identify individual differences that (a) predict better wellbeing outcomes after negative life experiences and (b) have high potential for use in interventions. Here, we examine whether the psychological impact of three negative life experiences—living with cystic fibrosis, being diagnosed with cancer, and going through a mass school shooting—is moderated by world beliefs.

Many people hold generalized beliefs about the world around them, sometimes called *primal* world beliefs to reflect their fundamental, general nature (e.g., “the world is beautiful”), in contrast to more specific or factual world beliefs (e.g., “the world is composed of chemicals”). These beliefs may be psychologically important, in that they shape people's expectations of the world. What's more, seeing the world as a more positive place—where people get treated fairly, where safety is the norm, where damaged things tend to heal, and where things can easily be improved—could be especially important at times of adversity, since people who hold these beliefs are more likely to see the negative events as an aberration from the (positive) norm, rather than as a sign of more negative things to come. Consistent with this hypothesis, people with more positive world beliefs tend to report better physical and mental wellbeing (Bartholomaeus and Strelan 2019; Clifton and Meindl 2022; Stahlmann et al. 2020).

One intuitive explanation for these correlations would be that positive events lead to both higher well‐being and more positive world beliefs, while negative events cause both lower well‐being and more negative world beliefs. Consistent with this, some trauma researchers have hypothesized that negative life experiences alter core components of individuals' world beliefs which in turn lead to negative psychological effects, including depression or anxiety (see *Shattered Assumption Theory*, Janoff‐Bulman 1989, 1992). These researchers theorized that some negative life experiences can lead to a sharp change in the belief that the world is benevolent or meaningful which, in turn, leads to declines in mental well‐being (e.g., Janoff‐Bulman 1992; Park and Folkman 1997; Tedeschi and Calhoun 2004). Indeed, there is some evidence to support the idea that world beliefs can play an important role in the negative psychological effects suffered by some as a consequence of traumatic events. For example, negative changes in world beliefs have been found to mediate the effect of trauma related to a shooting on depression (Littleton et al. 2012). Conversely, a review by Brown et al. (2019) found that changes in world beliefs (termed “world assumptions” in this work) mediated positive changes in the treatment of patients with PTSD.

However, while Shattered Assumptions Theory describes what *can* happen in some people, there is limited evidence that it describes the typical trajectory of most people who experience major negative life events (e.g., Lilly et al. 2015; Poulin and Silver 2019; Kerry et al. 2024). For example, even though Holocaust survivors see the world as less benevolent than demographically matched controls (Prager and Solomon 1995), the effect size was moderate even for this extreme experience. Further, various other studies found no link between several other major negative life experiences and beliefs that the world is good, safe, or just (e.g., Calhoun et al. 1998; Ginzburg 2004; Overcash et al. 1996; Tsang et al. 2025). For example, Kaler et al. (2008) found that increased lifetime trauma has only a small correlation with negative world beliefs and recent trauma did not influence existing beliefs. Similarly, recent research indicates that world beliefs are relatively stable over time and only weakly connected to a number of specific life events that people expect to have large effects on them (Clifton et al. 2019; Clifton 2020; Kerry et al. 2024; Ludwig et al. 2023). For example, dangerous world belief poorly reflects increased experiences of danger (e.g., living in a higher crime zip code, traumatic events in adulthood) and abundant world belief only weakly reflects personal experiences of abundance (e.g., being wealthy, living in a wealthy zip code, and growing up wealthy; Kerry et al. 2024). Longitudinal research has found that even major global events such as the outbreak of the Covid pandemic had little or no effect on people's world beliefs (Ludwig et al. 2023).

If world beliefs do not act as a mirror, reflecting life experiences, perhaps they act as a lens through which experiences are interpreted (Clifton 2020). According to Beck's (1967) cognitive model of depression, dysfunctional world beliefs could serve as interpretive schemas, which can explain the impact of stressful life events on negative thoughts, hopelessness, and depression by stimulating feelings of stress or anxiety in ambiguous situations and motivating inadequate health behaviors (Clifton and Kim 2020; Clifton and Meindl 2022). Consistent with this, dangerous world belief has been found to correlate with how people evaluate potential threats (Kerry and Clifton 2025). For example, people high in dangerous world belief (top quintile) tended to estimate national rates of such threats as murders, assaults, dog attacks, and robberies as several times higher (on average 4.2× higher) than people who scored the lowest. They also rated a range of activities (e.g., betting money, having unprotected sex, camping in the wilderness) as riskier, and described neutral‐faced strangers as less trustworthy, less happy, more threatening, and more likely to commit a crime.

If world beliefs change how people process new information, then it is possible that they could shape how people respond to adverse experiences. Perhaps people who see the world as a safe place where negative events are blips in an otherwise upward trend would be more likely to see a negative event as an aberration unlikely to be repeated. Some initial evidence suggests that world beliefs moderate the relationship between negative life experiences and negative well‐being outcomes: for example, bereaved people with stronger Just world belief experience lower levels of depression (Mancini et al. 2011). Similarly, a large cross‐sectional study found that young adults higher on Safe world belief—measured with Kaler's et al. (2008) World Assumptions Questionnaire—who reported having experienced interpersonal trauma had fewer depressive symptoms than those with lower Safe world belief (Schleider et al. 2021).

While these findings suggest that primal world beliefs may be promising moderators, the work so far on this topic is relatively sparse. Further, the lack of a more comprehensive measure of generalized world beliefs has meant that this work has been largely limited to examining three world beliefs: belief that the world is Good (or sometimes “benevolent” in assumptive world research), Safe, and Just. Further, researchers have often measured only one world belief at a time. Given that many world beliefs are substantially correlated (see Figure 1), this means that confounding cannot be ruled out. More broadly, many variables that are studied in relation to wellbeing and mental health are strongly valenced, and it can be hard to separate their effects from those of other variables that are strongly related to positive/negative feelings or outlook. Examples include optimism, hope, benevolent worldview, etc.

**FIGURE 1:**
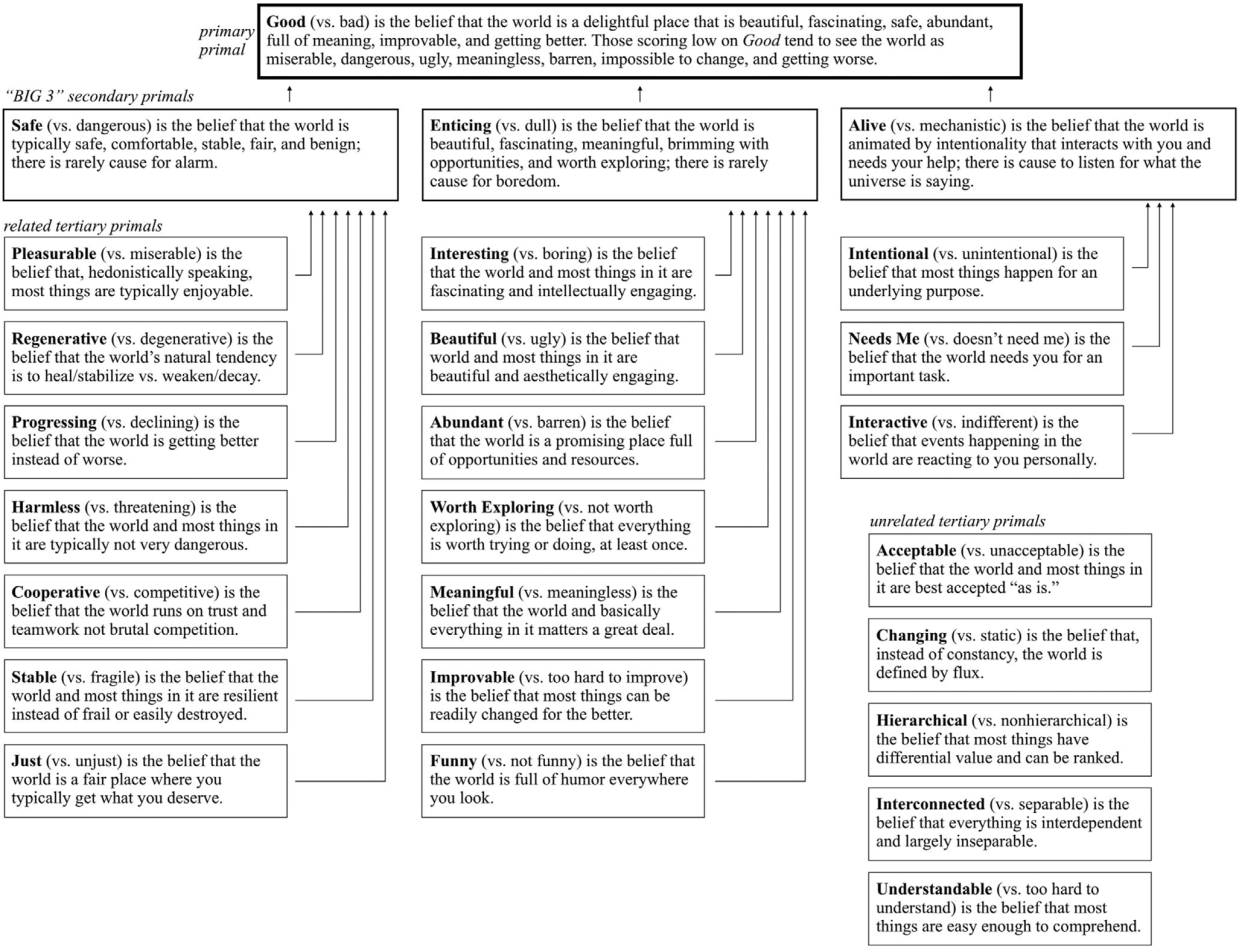
The hierarchical structure of primal world beliefs. *Source*: Clifton and Kim (2020).

To address some of these limitations in measurement, Clifton et al. (2019) set out to atheoretically develop a more comprehensive measure of world beliefs. They first captured an unprecedentedly broad set of inputs by analyzing thousands of tweets, historical texts, film scripts, etc., and received these inputs on a rolling basis until saturation was reached—months of searching vast datasets revealed no new candidate world beliefs. They then administered items to thousands of subjects and used factor analysis to reveal dimensionality. Results identified 26 important and distinct ways in which people disagree about the general nature of the world. As shown in Figure 1, the majority of these primals cluster into three secondary‐level world beliefs: Safe/dangerous, Enticing/dull, and Alive/mechanistic. In turn, these three secondary beliefs feed into the primary dimension: belief that the world is Good/bad. This comprehensive, structured, measure allows researchers to better discern between distinctive effects of specific world beliefs on relevant outcomes such as appraisals and wellbeing.

The present studies investigate the role of primal world beliefs in shaping emotional distress responses to severe negative life experiences. We hypothesized that, while overall positive beliefs about the world (i.e., Good world belief, which is the most closely related to optimism; *r* = 0.66 in Clifton et al. 2019) might act as buffers to some extent, world beliefs that were conceptually related to either the specific experience or to expectations of the future might be especially important.

We were interested in primals which gauge attitudes and expectations for the future and our ability to influence it. We hypothesized that for negative events related to threat, Safe world belief could act as an especially potent buffer. Safe world belief is theorized to inform expectations for the likelihoods of physical threats such as acts of violence or natural disasters (Kerry and Clifton 2025). Therefore, people lower in Safe world belief might be more likely to generalize acute experiences of physical danger, rather than treating extreme, threatening events as aberrations, unlikely to be repeated.

We also hypothesized relationships with beliefs that the world is Improvable (vs. too hard to improve), Just (vs. unjust), and Regenerative (vs. degenerative). This hypothesis was based on the logic that, when a devastating life event occurs, there are perhaps three routes for rectifying the situation: you (or another person) make it better, a higher power or karmic force intervenes, or the situation naturally gets better on its own. Improvable world belief holds that most things can be readily changed for the better—a plastic world amenable to change. People who believe in a Just world tend to think that good things happen to good people and that hard work and good behavior typically pay off in the end. Finally, individuals with high Regenerative world belief think that the world happens to be a place where the natural tendency of most things is to heal or to stabilize. In contrast, if one sees the world as a place where most circumstances resist human efforts to improve it, are chronically unfair, and naturally tend to devolve, decay, and fall apart, the arrival of great misfortune can signal that good times are permanently over and “it's all downhill from here”. Thus, we hypothesized that people higher in these three world beliefs would be less likely to experience long‐term increases in anxiety and depression as a consequence of highly negative events such as severe illness.

To test our hypotheses, we conducted two studies focusing on various groups who experienced severe negative life events. Study 1 examines four samples: current cancer patients, cancer survivors (i.e., people who were diagnosed with cancer but no longer experience symptoms), people with cystic fibrosis (a degenerative genetic illness affecting the respiratory system), and a control group. The aim was to test the hypothesis that world beliefs related to the potential for recovery and improvement—beliefs that the world is Improvable, Just, and Regenerative—moderate the relationship between severe illness and emotional distress.

We then tested whether similar moderation effects would be observed in a second, conceptually distinct context to assess whether the protective role of world beliefs extends beyond illness to acute, threatening events. Study 2 uses a longitudinal quasi‐experiment to examine effects over time, using data collected on a university campus before and after a mass shooting. It focuses on whether trait differences in Safe world belief moderate changes in stress experienced by students as a consequence of this tragic, potentially traumatic event, by examining this relationship both in the immediate aftermath and several months later.

## 1 Study 1

Study 1 aimed to test the moderating effects of world beliefs by examining four cross‐sectional samples: current cancer patients, cancer survivors, people living with cystic fibrosis, and a control group of healthy volunteers. In the case of current cancer patients and people with cystic fibrosis, participants at the time of the survey were still dealing with ongoing, chronic symptoms, and in many cases, a high risk of shortened lifespan. For comparison, we also included people with a history of cancer, but no current symptoms to test for longer‐lasting differences in psychological well‐being. Study 1 was not preregistered.

We hypothesized that beliefs that the world is improvable, just, and regenerative would moderate the relationship between having a severe illness and experiencing emotional distress. To test whether putative effects were better explained by a more generalized mechanism, such as having generally positive beliefs, we included higher‐order primal world beliefs—beliefs that the world is good, safe, enticing, and alive.

### 1.1 Method

All four samples (*N* = 1052) were collected concurrently over 6 months, with the aim of recruiting as many subjects as possible. All were unpaid volunteers who provided informed consent at the start of the survey. The study was approved by the University of Pennsylvania Institutional Review Board. No completed surveys were excluded.

#### 1.1.1 Participants

##### 1.1.1.1 Current Cancer Patients

We recruited 74 current cancer patients through Researchmatch.org (a website dedicated to matching people with specific medical conditions to research projects focusing on those conditions). The sample was 75.0% female, 23.8% male, and 1.2% intersex or other sex, and was aged 23–85 (*M* = 65.16, SD = 16.30).

##### 1.1.1.2 Cancer Survivors

We recruited 351 cancer survivors with no current symptoms from Researchmatch.org. The median time since recovery (i.e., most recent symptoms) was 6 years. The sample was 73.5% female, 23.4% male, and 1.1% intersex or other sex, and were aged 20–92 (*M* = 62.03, SD = 12.95).

##### 1.1.1.3 Control Group

The control sample comprised 484 US residents recruited through Researchmatch.org, who identified on this website as “healthy volunteers” who did not report any major physical or psychological illness. As part of our survey, these participants also confirmed that they had no history of cancer. This sample was 75.9% female, 23.0% male, 1.1% intersex/other, ages 20–88 (*M* = 48.16, SD = 17.24).

##### 1.1.1.4 Cystic Fibrosis

We recruited 117, mostly US‐based participants with cystic fibrosis via the Cystic Fibrosis Foundation (members were emailed by the foundation with information about the study). Participants were 80.3% female, 17.9% male, 0.9% intersex, 0.9% other or preferred not to say, and were aged 20–77 (*M* = 41.29, SD = 12.52).

### 1.2 Procedure

There was no experimental component, but survey order was randomized (*t*‐tests revealed no order effects on any world beliefs or well‐being measures, *p*'s > 0.05).

### 1.3 Measures

Descriptive statistics and reliabilities are displayed (both overall and within each sample) in Table S1. Reliabilities ranged from acceptable to excellent across most variables and samples. The exceptions were Safe (*α* = 0.67) and Just (*α* = 0.64) beliefs within the subsample of current cancer patients, which were slightly below the widespread cutoff of *α* = 0.70.

#### 1.3.1 World Beliefs

We used the Improvable, Regenerative, and Just world belief scales of the 99‐item Primals Inventory (PI‐99; Clifton et al. 2019). Participants rated agreement with items (from 0 = *Strongly disagree*; 5 = *Strongly Agree*). Improvable items include “It's possible to significantly improve basically anything encountered in life” and “Most situations seem really difficult if not impossible to improve” (reversed). Regenerative items include “The usual tendency of most things and situations is to get better, not worse” and “Most things have a habit of getting worse” (reversed). Just beliefs were assessed with five items including “The world is a place where working hard and being nice pays off” and “The world is a place where we rarely deserve what we get” (reversed) (improvable is considered a facet of Enticing, Regenerative and Just are facets of Safe—see Figure 1). It is worth noting that several primals have some conceptual overlap with other variables—Clifton et al. (2019) original validation paper includes extensive tests of discriminant and convergent validity for the world beliefs reported here.

We also used the Primals Inventory‐18 (PI‐18; Clifton and Yaden 2021) to measure the high‐order world beliefs: Good Safe, Enticing, and Alive, to test whether these could better explain relationships than the three focal beliefs listed above. No other world beliefs were measured. Reliability for all world belief scales was good (Cronbach's α > 0.78. See Table S1 for alphas by sub‐sample).

#### 1.3.2 Emotional Distress

Four items from the emotional distress item bank included in the Patient Reported Outcome Measurement Information System (PROMIS—Pilkonis et al. 2011) were used to measure depression and four measured anxiety. Participants indicated how often they experienced a series of symptoms over the last week (from 1 = *never*, 5 = *always*), such as “In the past 7 days I felt helplessness” (depression) and “In the past 7 days I felt fearful” (anxiety). We combined the measures into one emotional distress variable, since they were closely correlated (*r* = 0.86).

### 1.4 Study 1 Results and Discussion

Correlations between variables are shown in Table S2. Data and code for Studies 1 and 2 can be accessed at: https://osf.io/bjrc8/?view_only=5e1394310fce49da9a2160a142cc3a73.

#### 1.4.1 Preliminary Analyses

**FIGURE 2:**
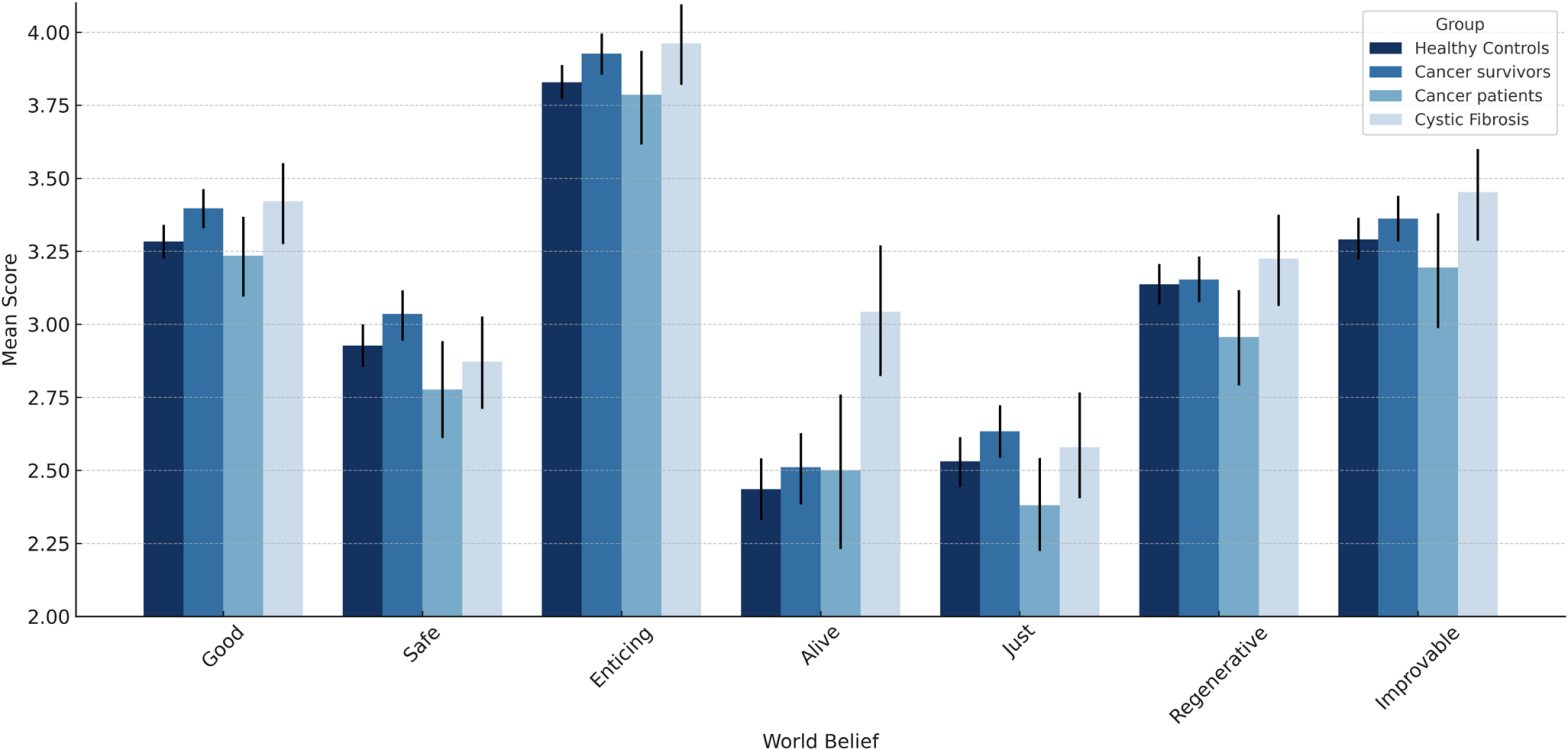
Group means for all measured world beliefs.

**FIGURE 3:**
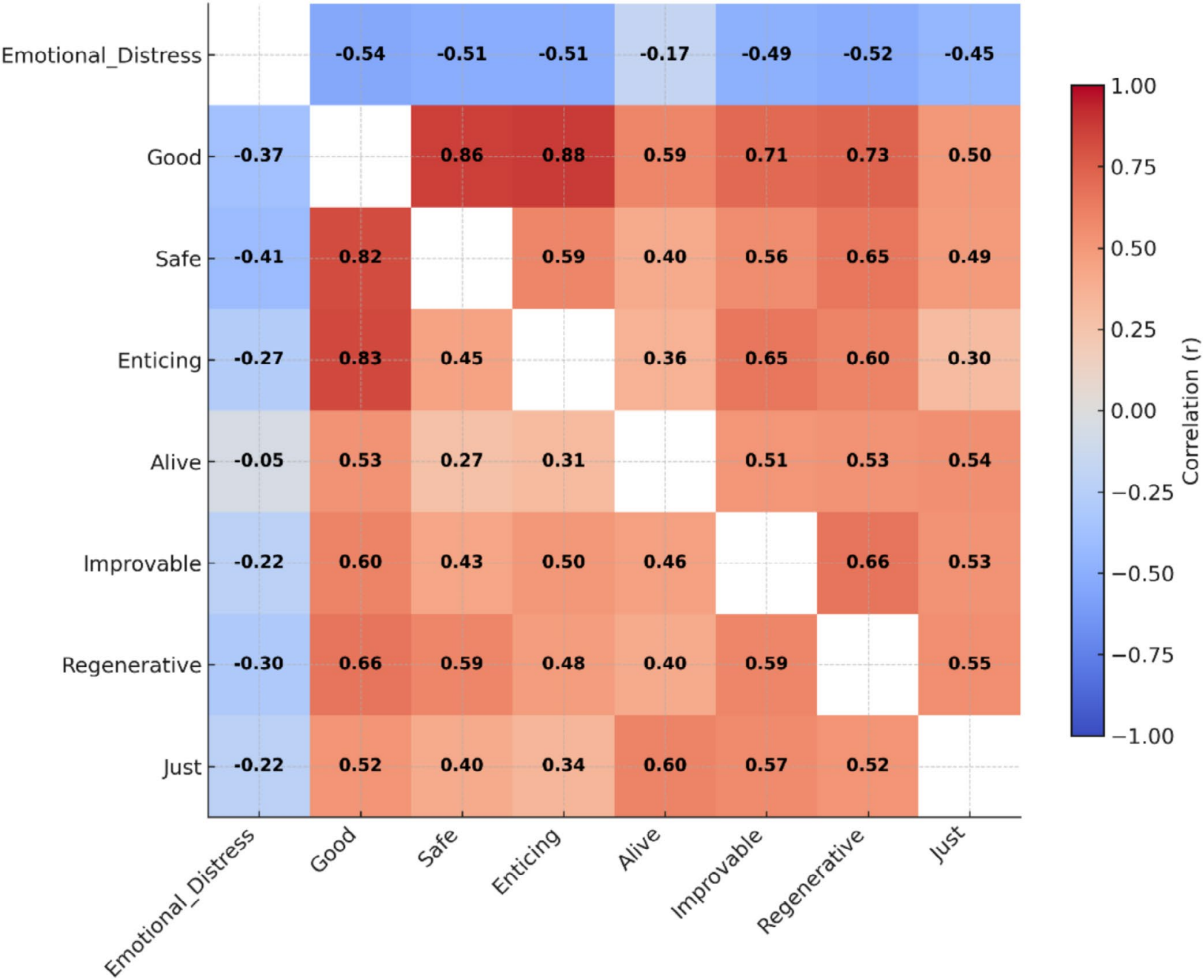
Correlations among people with current illness (top right) and a healthy control group (bottom left).

There were no overall differences between groups on most world beliefs. For example, people with cancer and cystic fibrosis scored no differently overall on most world beliefs compared to the control group. The notable exception was that people with Cystic Fibrosis scored substantially *higher* than controls on the belief that the world is Alive (i.e., full of intention and meaning), while people with a current cancer diagnosis scored slightly lower than controls on Regenerative and Just beliefs. Group means are shown in Figures 2 and 3 shows overall correlations between main variables, while Tables S2–S6 show both combined and within‐subsample correlations.

There were no differences in emotional distress between cancer survivors and controls, either in terms of the overall difference or moderated effects. Notably, there was a modest negative correlation among cancer survivors between time since recovery and levels of emotional distress (*r* = −0.15, *p* = 0.006), consistent with the idea of a gradual return to baseline levels across a period of several years (the median time since recovery in this sample was 6 years). The following moderation analyses therefore focus on participants with ongoing health problems.

#### 1.4.2 Primal World Beliefs Moderated the Link Between Severe Illness and Emotional Distress

**FIGURE 4:**
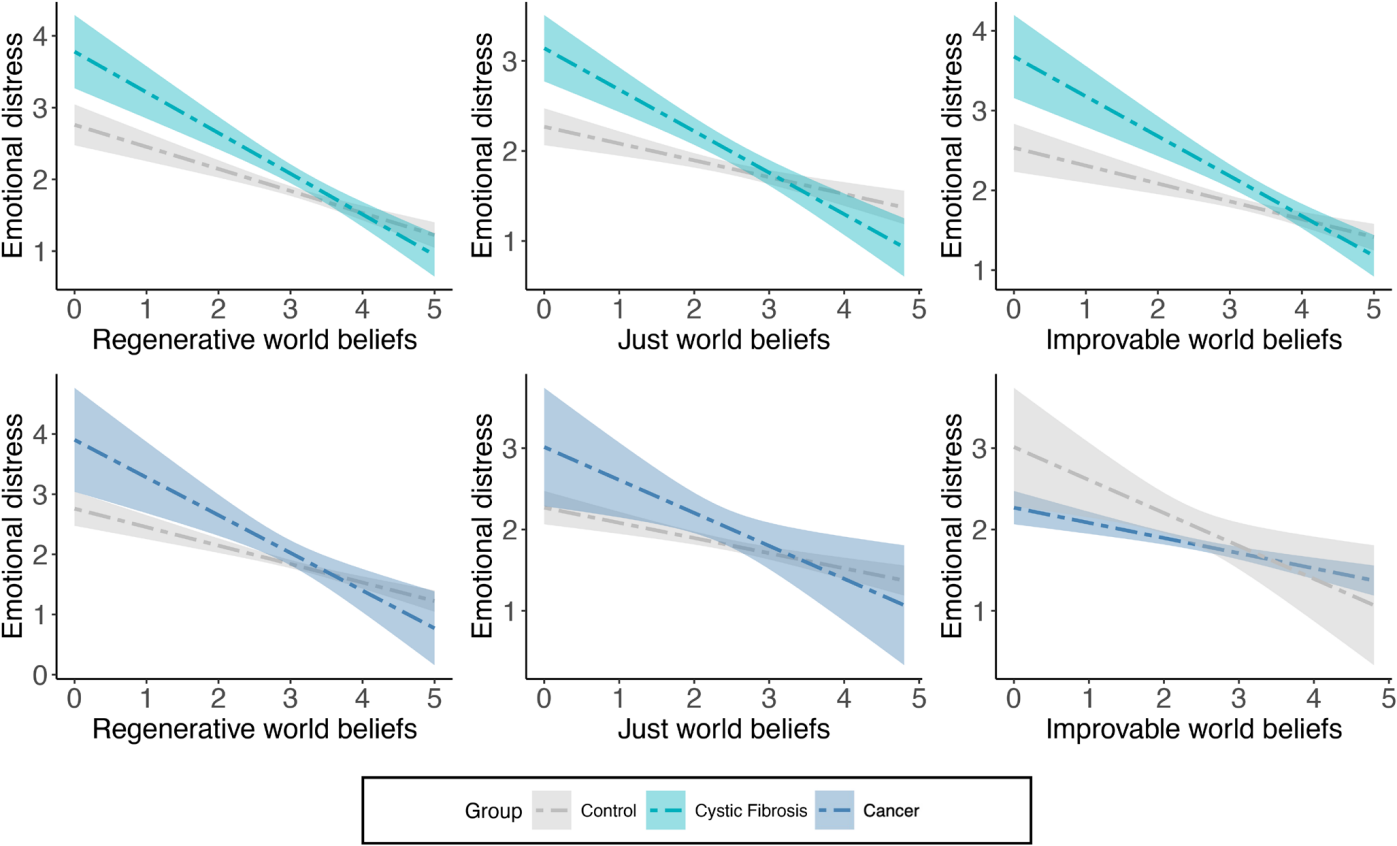
Regression plots illustrating the moderating role of regenerative world Beliefs. *Note*: Colored bands denote 95% confidence intervals.

Primal world beliefs, especially the focal beliefs that the world is Improvable, Just, and Regenerative, substantially moderated the link between the negative life events and emotional distress (See Figure 4 and Table 2). Among people who saw the world as more Improvable, Regenerative, and Just, there was no relationship between having a chronic illness and reporting symptoms of emotional distress, but there were moderate‐to‐large differences among people with lower levels of these beliefs.

To simplify analyses and increase power (and since patterns of results for both groups were very similar), we created a combined current illness dummy variable which included both current cancer patients and cystic fibrosis patients (*n* = 192)—compared to the control group (*n* = 501). The main analyses below report moderated effects for this combined variable in relation to the control group. Because we ran several moderated regression models for different beliefs, we chose a lower alpha threshold of *p* < 0.01 for these main analyses.

Three moderated regression analyses using the combined current illness variable as a predictor alongside a world belief moderator variable and their interaction term, with emotional distress as the dependent variable, found strong moderation effects of Improvable (*b* = −0.27, 95% CI [−0.42, −0.12], *p* < 0.001), Just (*b* = −0.29, 95% CI [−0.45, −0.13], *p* < 0.001), and Regenerative (*b* = −0.27, 95% CI [−0.43, −0.11], *p < 0*.001) beliefs. In all three analyses, differences between the current illness group and controls at high levels (+1 SD) of the world belief were nonsignificant (all *p's* ≥ 0.75). Conversely, in all three analyses, at low levels (−1 SD) of Improvable, Just, and Regenerative beliefs there was a strong positive association between current illness and emotional distress (all *b*'s > 0.39, *p*'s < 0.001).

An alternative way to describe these moderations is that the negative relationship between each of these primal world beliefs and emotional distress was about twice as strong among people with serious illnesses than among people in the healthy control group. For example, the relationship between Regenerative belief and emotional distress was *r*(480) = −0.30 [−0.38, −0.22], *p* < 0.001, among healthy controls (or 9% variance explained), while the correlation among people with cancer or cystic fibrosis was *r*(178) = −0.52, 95% CI [−0.62, −0.41], *p* < 0.001, (or 27% shared variance).

Results were consistent across illness type (see Table 1). Controlling for age and sex made little difference and, if anything, made interaction effects slightly stronger (see Table S7).

**TABLE 1:** Moderated associations between severe current illness and emotional distress.

Group	Moderator	Slope at −1 SD	Slope at +1 SD	Interaction *p*
Cancer patients	Improvable	0.40**	0.02	0.024*
Cystic fibrosis	Improvable	0.43***	0.04	0.002**
**Combined**	**Improvable**	0.44***	**0.00**	< 0.001***
Cancer patients	Just	0.40**	0.00	0.090
Cystic fibrosis	Just	0.43***	−0.06	< 0.001***
**Combined**	**Just**	0.44***	**−0.03**	< 0.001***
Cancer patients	Regenerative	0.35**	−0.13	0.016*
Cystic fibrosis	Regenerative	0.37***	−0.02	0.006**
**Combined**	**Regenerative**	0.40***	**−0.03**	< 0.001***

*Note:* *p < 0.05. **p < 0.01. ***p < 0.001. Combined values are bolded.

Abbreviation: SD = standard deviations of the moderator.

Further, adjusting the analyses such that cancer survivors were also included in the control group (thus including all participants in the analysis) made little difference to results (interactions for Regenerative, Improvable, and Just all *p* < 0.001).

#### 1.4.3 Effects Were Not Better Explained by Good World Belief

The association between severe illness and emotional distress was moderated by overall beliefs that the world is Good (*b* = −0.20, 95% CI [−0.39, −0.02] *p* = 0.033), but this moderation effect was weaker than for the three focal world beliefs reported above. Analysis of the secondary‐level primals suggested that this effect was largely driven by Enticing belief (*b* = −0.47, 95% CI [−0.47, −0.11], *p* = 0.002)—which includes the more specific beliefs that the world is Interesting, Beautiful, Meaningful, and Improvable—while Safe (*b* = −0.13, 95% CI [−0.29, 0.02], *p* = 0.089) and Alive (*b* = −0.09, 95% CI [−0.20, 0.02], *p* = 0.125) were nonsignificant moderators. Although Good and Enticing belief were statistically significant moderators, neither performed as well as the three focal lower‐order beliefs that we included: belief that the world is, Improvable, Just, and Regenerative. Models which included any of these beliefs and controlled for overall Good world belief and its interaction terms invariably yielded a significant moderation effect for the lower‐order world belief, but not for Good.

## 2 Study 2

On November 13, 2022, a shooter opened fire on the campus of the University of Virginia (UVA), killing three students and injuring two more. While authorities searched for the perpetrator, a shelter‐in‐place order was in force across the campus for roughly 24 h, after which classes were canceled for two further days. In addition to the tragic effects of this event on the victims and people close to them, the shooting represented a disturbing experience for many others on campus, with high potential to affect emotional distress and mental health.

Study 2 involved the unplanned use of data from before and after this shooting. This data was collected on the UVA campus for another project (the researchers involved in Study 1 approached the UVA team with the intention of testing the hypotheses presented here). Study 2 thus builds on the results of Study 1 by examining the buffering effects of world beliefs for a different type of negative live event—being in the vicinity of a mass shooting. Importantly, this naturalistic and longitudinal data allowed us to test the effects of a concrete real‐life event and examine within‐person changes over time.

The originally planned study had aimed to test the relationship between Safe and Enticing beliefs and students' movement patterns and included a measure of the four superordinate primal world beliefs: Good, Safe, Enticing, and Alive. Given that the shooting has obvious implications for the safety of the local environment, we hypothesized that Safe world belief would be the most important moderator. However, we also report analyses for the other measured world beliefs as a more rigorous test of discriminant validity.

Due to the unplanned nature of the study, pre‐registration was not possible, and the lower‐level beliefs included in Study 1 (Regenerative, Improvable, Just) were not measured.

### 2.1 Method

#### 2.1.1 Participants and Recruitment

152 participants (*Mage* = 19.73, SD = 1.32; 35 men, 95 women, 15 non‐binary, 5 preferred not to describe, 2 other; 71 White/Caucasian, 47 Asian, 17 Mixed‐race, 8 Black or African American, 4 Hispanic or Latino, 3 Middle Eastern, 1 Native Hawaiian or Other Pacific Islander, 1 Other) were recruited from three residential colleges within the university for the purpose of a study examining the relationship between participants' beliefs and physical movement patterns.

The study was advertised as an online, multi‐part study, where participants could complete the 2‐week behavior tracking component, the survey component, both, or either. Participants were paid $20 for completing each mobile sensing component, and $10 for completing each survey component. The study was approved by the University of Virginia's IRB. Informed consent was obtained via electronic signature at the start of the study.

### 2.2 Procedure

Surveys were completed in three waves: in September 2022, November 2022, and March 2023. In each wave, there was a 2‐week behavioral tracking component followed by a 15‐min survey component. In the second wave, the campus shooting occurred directly in the middle of the 2‐week behavioral tracking component (Day 7 of 14), and the survey component was administered 9 days after the shooting. Students completed the Wave 2 survey between the dates of November 22nd and November 30th. The daily‐tracking component of the study collected ecological momentary assessments and location data. However, this information is not relevant to the current hypotheses and is omitted for brevity.

#### 2.2.1 Attrition

In total, 115 participants completed the survey for at least two waves. To determine whether key variables predicted attrition, we regressed Wave 2 and Wave 3 missingness on participants' Wave 1 average beliefs that the world is Safe, Enticing, and Alive, as well as perceived stress. None of these predicted whether participants dropped out for Waves 2 or 3, suggesting that the effects of Wave in our analyses are not due to nonrandom attrition.

### 2.3 Measures

#### 2.3.1 Primal World Beliefs

Beliefs that the world is Safe, Enticing, and Alive were measured with the PI‐18 scale, as in Study 1 (Clifton and Yaden 2021). No other world beliefs were measured (since the originally planned study was just focused on Safe and Enticing world beliefs). Because there was no evidence that these beliefs changed in response to the shooting (see results section below) and to minimize measurement error, we created a trait score for each belief by mean‐scoring across the three timepoints. Primals scores showed a high degree of stability, with the test–retest correlations across the three waves ranging from *r* = 0.68 to *r* = 0.88. Reliabilities were good across all world beliefs (all Cronbach's α's > 0.75).

#### 2.3.2 Perceived Stress

Perceived stress was measured with the 10‐item Perceived Stress Scale (Cohen et al. 1983), which includes items such as “In the last month, how often have you felt nervous and “stressed”?” and “In the last month, how often have you felt difficulties were piling up so high that you could not overcome them?”. Items are rated on a five‐point frequency scale (1 = never; 2 = almost never; 3 = sometimes; 4 = fairly often; 5 = very often). Reliability was good Cronbach's α = 0.82.

#### 2.3.3 Other Measures

Participants also answered surveys on several measures. However, these did not include other measures that could plausibly be used to test the present hypotheses.

### 2.4 Study 2 Results and Discussion

**FIGURE 5:**
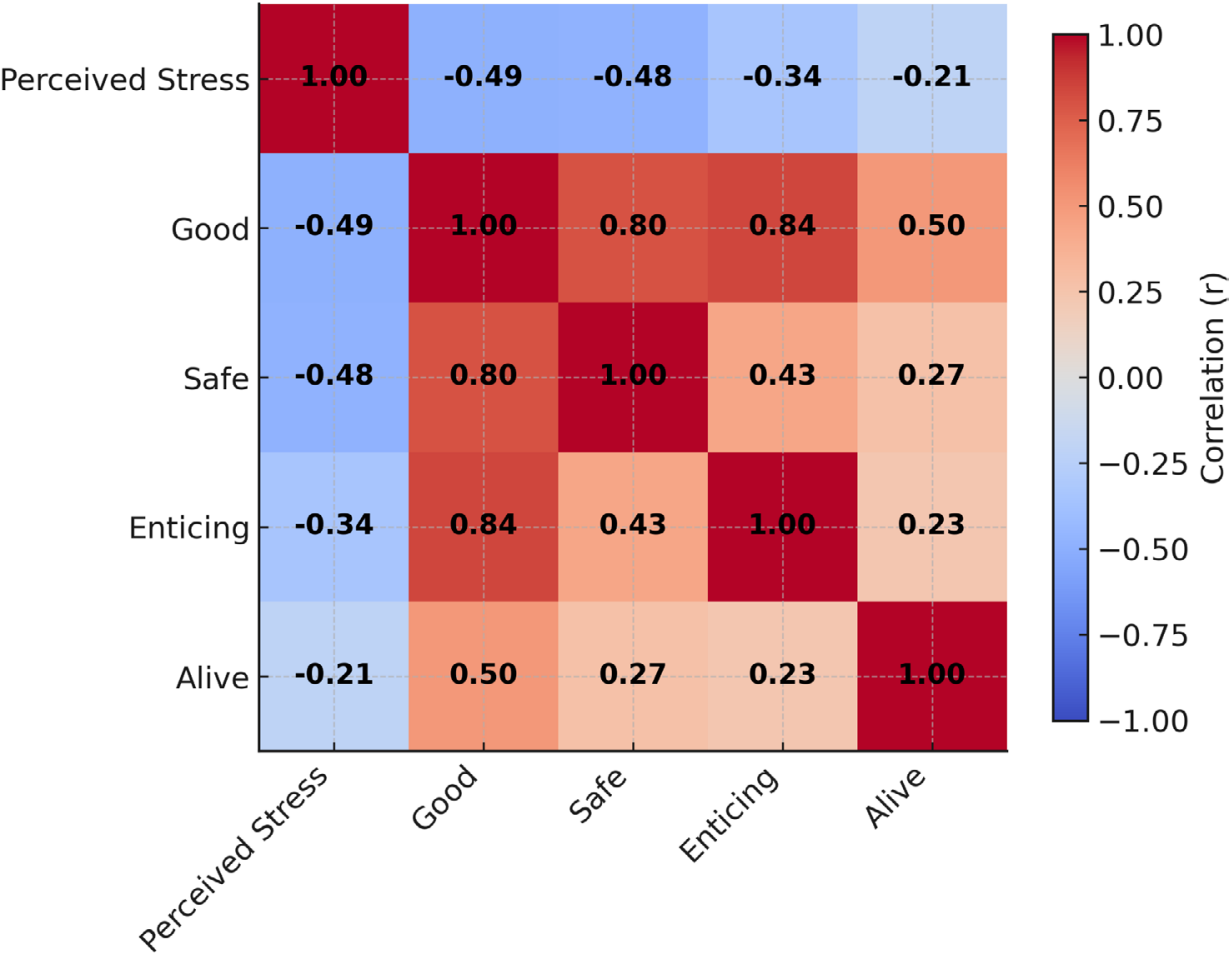
Correlations between main variables in Study 2.

Overall correlations between variables are shown in Figure 5 (within‐wave correlations are shown in Figure S1).

#### 2.4.1 Overall Effects of the Shooting on World Beliefs or Stress Levels

None of the focal world beliefs changed across the three waves, consistent with previous research suggesting that world beliefs have a high degree of stability, even after potentially stressful events (e.g., Clifton et al. 2019; Kerry et al. 2024; Ludwig et al. 2023). A series of paired sample *t*‐tests revealed no differences between scores at Wave 1 and those at either Wave 2 or Wave 3. The only comparisons that approached significance were in the opposite direction of what might be expected, i.e., people reported seeing the world as nonsignificantly Safer (*d* = 0.22, *p* = 0.072) and more Enticing (*d* = 0.22, *p* = 0.073) at Wave 3 versus Wave 1 (for all other comparisons *d*'s ≤ 0.09, *p*'s ≥ 0.46). Table 2 shows means by group.

**TABLE 2:** Means by wave and ANOVAs of differences.

Variable	Wave 1 M	Wave 2 M	Wave 3 M	ANOVA	W1 vs. W2	W2 vs. W3	W1 vs. W3
Perceived stress	2.98 (0.64)	3.27 (0.67)	3.11 (0.70)	*p = 0*.002	*p* = 0.002	*p* = 0.146	*p* = 0.277
Good	3.11 (0.59)	3.02 (0.54)	3.08 (0.65)	*p* = 0.464	*p* = 0.553	*p* = 0.666	*p* = 0.949
Safe	2.36 (0.69)	2.30 (0.70)	2.49 (0.80)	*p* = 0.789	*p* = 0.791	*p* = 0.861	*p* = 0.931
Enticing	3.67 (0.70)	3.58 (0.66)	3.64 (0.75)	*p* = 0.601	*p* = 0.572	*p* = 0.836	*p* = 0.911
Alive	3.08 (1.11)	2.94 (1.11)	3.07 (1.10)	*p* = 0.561	*p* = 0.518	*p* = 0.641	*p* = 0.999

Self‐perceived stress increased in the immediate aftermath of the shooting before returning to baseline: Among students who participated in at least two waves, stress was higher at Wave 2 versus Wave 1 (3.22 vs. 2.99, *d* = 0.37, *t* = 3.22, *p* = 0.002), but was not significantly different from Wave 1 and Wave 3 (3.03 vs. 3.01, *d* = 0.03, *t* = 3.22, *p* = 0.784). Consistent with earlier work examining wellbeing trajectories before and after a campus shooting (Mancini et al. 2016), we found substantial individual differences, such that not all students reported increased stress levels, even shortly after the shooting. Of 74 participants who reported stress levels at Wave 2, 44 showed an increase, 6 reported identical stress levels, and 24 showed a decrease. For the 68 who reported at Wave 1 and Wave 3, 35 showed an increase, 3 showed no movement and 30 showed a decrease in stress.

#### 2.4.2 Safe World Belief Moderated the Effects of the Shooting on Students' Stress Levels

To test whether trait‐level Safe belief predicted more positive trajectories in terms of changes in stress levels, we ran a mixed‐effects regression model in which a within‐person mean score for Safe world belief, a dummy code representing each wave, and two wave*world belief interaction terms were entered as fixed effects, with participant ID as a level 2 grouping variable for which the intercept was allowed to vary at random.

**FIGURE 6:**
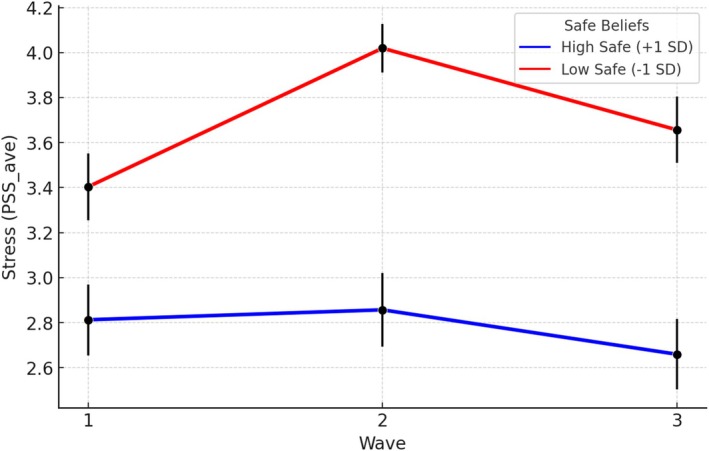
Safe world belief moderates the effects of a campus shooting on stress. *Note*: Vertical bars represent standard errors. Safe beliefs are trait scores (averaged across waves). Shooting occurred after W1 and 9–17 days before W2 data collection.

Consistent with Study 1, world beliefs appeared to predict who would experience elevated stress: trait‐level Safe world beliefs significantly moderated the relationship between Wave and Perceived Stress when comparing Wave 1 (before shooting) to either Wave 2 (collected 9–17 days after the shooting), *b* = −0.26 (95% CI [−0.49, −0.03], *p* = 0.025), or Wave 3, (4 months after the shooting), *b* = −0.29 (95% CI [−0.50, −0.08], *p* = 0.007). Mirroring the pattern of interactions observed in cross‐sectional data in Study 1, people high in trait‐level Safe world beliefs reported no increase in self‐perceived stress after the shooting, while people lower in Safe world beliefs showed a substantial increase (see Figure 6).

One possible explanation for this finding is that the moderation effects were driven by having generally positive world beliefs, rather than relating to Safe beliefs in particular. To better test the discriminant validity of our hypothesis, we ran a series of analyses with other positively valenced world beliefs as moderators. Belief that the world is Good did not moderate this relationship (Tables S8–S11 show moderated regressions for each of the four world beliefs separately). Further, when all three secondary‐level primals and their interaction terms were included in the same model to account for shared variance (i.e., Safe, Enticing, and Alive), only Safe emerged as a significant moderator (see Table 3). Thus, the moderation by Safe belief does not simply come down to holding generally positive beliefs.

**TABLE 3:** Mixed effects regression with all secondary primals and interaction effects.

Predictors	PSS_ave		
	Estimates	CI	*p*
Intercept	3.08	2.98 to 3.18	**< 0.001**
Safe (trait)	−0.30	−0.48 to −0.11	**0.002**
After shooting (dummy)	0.17	0.06 to 0.29	**0.003**
Enticing (trait)	−0.23	−0.40 to −0.05	**0.011**
Alive (trait)	0.00	−0.10 to 0.10	0.998
Safe X after shooting	−0.30	−0.52 to −0.08	**0.008**
Enticing X after shooting	0.13	−0.08 to 0.35	0.207
Alive X after shooting	−0.08	−0.20 to 0.03	0.147
**Random effects**			
*σ* ^2^	0.20		
τ_00 userID_	0.18		
ICC	0.47		
*N*_userID_	116		
Observations	286		
Marginal *R* ^2^/Conditional *R* ^2^	0.217/0.586		

*Note:* After Shooting (dummy) variable represents a contrast W1 with W2 and W3 combined. Bolding indicates statistically significant effects.

### 2.5 Robustness

In the above analyses, we chose to aggregate primal measures across time points to increase the reliability of the trait estimate by reducing random measurement error, and to enhance statistical power by allowing us to include all data. However, we should note that running a post hoc robustness check where only the Time 1 measure of Safe was used led to a directionally consistent but statistically nonsignificant interaction, *b* = −0.13, 95% CI [−0.32, 0.06], *p* = 0.187. Thus, while the current results are largely consistent with our main hypothesis (and with some existing research—e.g., Mancini et al. 2011), further research is important to establish whether *prior* trait levels of world beliefs are prospectively predictive.

## 3 General Discussion

Why do some people experience steep increases in emotional distress after major negative life experiences, while others do not? Study 1 explored this question by comparing groups who experienced severe illness with a healthy control group. We found that among people who viewed the world as more improvable, just, and regenerative, levels of anxiety and depression were no higher than in controls. In contrast, among people lower in these beliefs, chronic illness was associated with substantially higher emotional distress. Study 2 provided parallel evidence in a longitudinal sample: students with higher trait levels of safe world belief showed no increases in stress following a mass shooting on their university campus, whereas students lower in safe belief reported significant and lasting increases in stress. Taken together, these two studies provide converging evidence that world beliefs can buffer distress across highly different forms of adversity—chronic illness and acute trauma—and across timescales ranging from weeks to years.

The finding that world beliefs moderate distress effects of major negative experiences is consistent with research suggesting that bereaved people with stronger Just World belief experience fewer symptoms of depression than those with lower levels (Mancini et al. 2011). Our findings conceptually replicate this earlier finding and also suggest that (a) the effect may be generalizable to other negative experiences, (b) this effect is largely independent of related beliefs, and (c) some other world beliefs—especially Safe, Regenerative, and Improvable world beliefs—may be equally or more important. Thus, the findings here offer corroboration for these earlier findings, in samples with high ecological validity. Taken together, this research suggests that, while world beliefs have often been studied as potential mediators of the effects of negative experiences on emotional distress (Janoff‐Bulman 1992; Park and Folkman 1997; Tedeschi and Calhoun 2004), trait‐like individual differences in these beliefs may be useful in explaining why some people are more likely to suffer emotional distress after adverse events than others.

An important limitation concerns the discriminant validity of the world belief constructs examined. Although Improvable, Regenerative, and Just beliefs are conceptually and empirically distinct, they are substantially intercorrelated, as is typical for positively valenced psychological constructs (Clifton et al. 2019). This raises the possibility that some of the observed effects could reflect shared variance among these beliefs rather than unique contributions of each construct. It is also possible that the moderating effect of these world beliefs could be equally well explained by a more well‐established measure such as hope or optimism. However, several points suggest greater specificity. First, moderation effects differed across beliefs (e.g., Safe belief was a stronger moderator of stress after physical threat than other positive world beliefs in Study 2). Second, in Study 1, higher‐order beliefs such as Good and Enticing were weaker moderators than the more specific beliefs of interest, and including these as covariates did not eliminate effects. This is particularly pertinent because, in previous studies, Good was more strongly correlated to optimism than any other world belief (*r* = 0.66—Clifton et al. 2019). Nonetheless, we encourage future work to more directly examine discriminant validity.

Even if optimism (or a similar construct) also moderates the impact of negative life experiences, world beliefs may nonetheless represent a promising target for interventions. First, there are strong theoretical reasons to think that world beliefs may be causal priors to personality‐like traits such as optimism and hope (Clifton and Crum 2024). Second, it is possible that beliefs are more easily manipulated through cognitive‐ and information‐based interventions than dispositional measures such as personality traits and character strengths (Dweck 2008). Indeed, although interventions targeting primal world beliefs are in their infancy, some initial work has found evidence of their malleability (Hämpke et al. 2024; Snook et al. 2025). Thus, future interventions may do well to test Safe, Regenerative, Improvable, and Just beliefs as more proximate and malleable pathways to improved wellbeing outcomes.

Several important limitations remain, such that future work is required to more fully understand these relationships. Given that neither study reported here conclusively showed an earlier world belief predicting later change, further studies and meta‐analyses may be necessary for greater confidence about causal relationships. Further, although we saw some indications in these studies that particular world beliefs might be more important for specific types of negative experience, there is a clear need to test whether this specificity can be reliably demonstrated.

## 4 Concluding Remarks

In difficult times, it matters how one interprets uncertainty. Primal world beliefs are theorized to play a greater role in interpreting any given situation the more uncertain the situation becomes. Specifically, humans vary in their belief that most circumstances in the world are safer versus more dangerous (Safe world belief), amenable to change versus too hard to improve (Improvable world belief), fair versus unfair (Just world belief), and whether the natural principle of the universe is to heal and stabilize versus decay and fall apart (Regenerative world belief). We found evidence that when one holds these four beliefs, highly negative life experiences are less likely to result in anxiety, or depression. In contrast, when one sees the world as dangerous, too hard to improve, unfair, and prone to degenerate, great difficulty drives long‐term struggle. This suggests a buffering role of some world beliefs. Future research might explore if interventions to recalibrate these beliefs could improve wellbeing trajectories during times of illness, personal tragedy, natural disaster, and war.

## Author Contributions

Conceptualization: N.K. and J.D.W.C.; Data collection: All authors; Statistical analyses and cleaning: N.K., A.W., J.H., and S.T.; Figures: N.K., J.H., and A.W.; Writing (main): N.K., J.H., and J.D.W.C.; Writing (minor edits and approval of manuscript): All authors.

## Ethics Statement

Both studies were approved by the relevant institutional review board (Study 1—University of Pennsylvania; Study 2—University of Virginia).

## Conflicts of Interest

The authors declare no conflicts of interest.

## Supporting information


**Data S1:** Supporting information.

## Data Availability

Anonymized data for these studies and code for the main analyses are accessible at: https://osf.io/bjrc8/?view_only=5e1394310fce49da9a2160a142cc3a73. The studies reported here were not preregistered.
