# Supplementary material for: World Beliefs Moderate the Effects of Trauma and Severe Illness on Emotional Distress
Source: J Pers. 2025 Nov 12;94(4):690–702. doi: 10.1111/jopy.70031 (PMC13359294; doi:10.1111/jopy.70031)
Supplement: Supplementary file 1 — Data S1: Supporting information. [file JOPY-94-690-s001.docx]

**Supplemental Material**

**Table S1**

*Descriptive Data and Reliabilities in Study 1 (Cronbach’s α)*

|  | Cancer  (*n* = 74) | | | Cancer survivor  (*n* = 351) | | | Cystic fibrosis  (*n* = 117) | | | Control group  (*n* = 484) | | | | Overall  (*N* =1,026) | | | |
| --- | --- | --- | --- | --- | --- | --- | --- | --- | --- | --- | --- | --- | --- | --- | --- | --- | --- |
|  | *M* | *SD* | **α** | *M* | *SD* | **α** | *M* | *SD* | **α** | *M* | *SD* | **α** | *M* | | *SD* | **α** |  |
| Good | 3.24 | 0.62 | .84 | 3.40 | 0.62 | .85 | 3.42 | 0.74 | .91 | 3.28 | 0.60 | .86 | 3.32 | | 0.64 | .85 |  |
| Safe | 2.78 | 0.73 | .67 | 3.04 | 0.83 | .82 | 2.87 | 0.88 | .85 | 2.93 | 0.76 | .80 | 2.95 | | 0.80 | .81 |  |
| Enticing | 3.79 | 0.70 | .77 | 3.93 | 0.65 | .77 | 3.96 | 0.75 | .86 | 3.83 | 0.65 | .78 | 3.87 | | 0.66 | .79 |  |
| Alive | 2.50 | 1.17 | .85 | 2.51 | 1.17 | .85 | 3.04 | 1.22 | .89 | 2.44 | 1.15 | .86 | 2.54 | | 1.18 | .86 |  |
| Improvable | 3.19 | 0.86 | .85 | 3.36 | 0.77 | .79 | 3.45 | 0.90 | .86 | 3.29 | 0.78 | .83 | 3.32 | | 0.81 | .83 |  |
| Regenerative | 2.96 | 0.73 | .77 | 3.15 | 0.74 | .78 | 3.22 | 0.83 | .81 | 3.14 | 0.77 | .83 | 3.13 | | 0.78 | .81 |  |
| Just | 2.38 | 0.76 | .64 | 2.63 | 0.87 | .76 | 2.58 | 0.98 | .83 | 2.53 | 0.91 | .81 | 2.56 | | 0.90 | .79 |  |
| Depression | 2.11 | 1.03 | .92 | 1.82 | 0.85 | .88 | 2.11 | 0.87 | .90 | 1.89 | 0.84 | .91 | 1.79 | | 0.89 | .91 |  |
| Anxiety | 1.99 | 1.05 | .90 | 1.71 | 0.83 | .89 | 1.79 | 0.90 | .87 | 1.71 | 0.82 | .88 | 1.97 | | .91 | .90 |  |
| Emotional Distress | 2.05 | 1.00 | .95 | 1.77 | 0.81 | .93 | 1.95 | 0.84 | .93 | 1.80 | 0.79 | .94 | 1.82 | | 0.82 | .94 |  |

**Table S2**

*Bivariate Correlations in Study 1 Combined Sample (n=1,028)*

|  | 1 | 2 | 3 | 4 | 5 | 6 | 7 | 8 | 9 |
| --- | --- | --- | --- | --- | --- | --- | --- | --- | --- |
| 1. Emotional Distress | — |  |  |  |  |  |  |  |  |
| 2. Regenerative | -.33** | — |  |  |  |  |  |  |  |
| 3. Improvable | -.28** | .58** | — |  |  |  |  |  |  |
| 4. Good | -.40** | .67** | .62** | — |  |  |  |  |  |
| 5. Safe | -.43** | .60** | .47** | .83** | — |  |  |  |  |
| 6. Enticing | -.32** | .50** | .51** | .83** | .47** | — |  |  |  |
| 7. Alive | -.03 | .42** | .43** | .54** | .29** | .28** | — |  |  |
| 8. Just | -.25** | .50** | .53** | .48** | .42** | .27** | .53** | — |  |
| 9. Age | -.31** | .08* | .10** | .21** | .23** | .18** | -.03 | -.15** | — |
| 10. Sex | .08* | .01 | .02 | .08* | .09** | .09** | .13** | -.04 | -.21** |

**Within-groups correlations in Study 1.**

**Table S3**

*Correlations in Control Group (Study 1)*

|  | Good | Safe | Enticing | Alive | Improvable | Regenerative | Just |
| --- | --- | --- | --- | --- | --- | --- | --- |
| Emotional Distress | -0.37*** | -0.41*** | -0.27*** | -0.05 | -0.22*** | -0.30*** | -0.22*** |
| Good | — | 0.82*** | 0.83*** | 0.53*** | 0.60*** | 0.66*** | 0.52*** |
| Safe |  | — | 0.45*** | 0.27*** | 0.43*** | 0.59*** | 0.40*** |
| Enticing |  |  | — | 0.31*** | 0.50*** | 0.48*** | 0.34*** |
| Alive |  |  |  | — | 0.46*** | 0.40*** | 0.60*** |
| Improvable |  |  |  |  | — | 0.59*** | 0.57*** |
| Regenerative |  |  |  |  |  | — | 0.52*** |
| Just |  |  |  |  |  |  | — |

**Table S4**

*Correlations among Cancer Survivors (Study 1)*

|  | Good | Safe | Enticing | Alive | Improvable | Regenerative | Just |
| --- | --- | --- | --- | --- | --- | --- | --- |
| Emot. Distress | -0.33*** | -0.40*** | -0.26*** | 0.04 | -0.22*** | -0.25*** | -0.18*** |
| Good | — | 0.84*** | 0.79*** | 0.51*** | 0.57*** | 0.66*** | 0.42*** |
| Safe |  | — | 0.43*** | 0.29*** | 0.47*** | 0.59*** | 0.41*** |
| Enticing |  |  | — | 0.20*** | 0.41*** | 0.45*** | 0.15** |
| Alive |  |  |  | — | 0.34*** | 0.41*** | 0.45*** |
| Improvable |  |  |  |  | — | 0.52*** | 0.46*** |
| Regenerative |  |  |  |  |  | — | 0.44*** |

**Table S5**

*Correlations among Cancer Patients (Study 1)*

|  | Good | Safe | Enticing | Alive | Improvable | Regenerative | Just |
| --- | --- | --- | --- | --- | --- | --- | --- |
| Emotional Distress | -0.50*** | -0.40*** | -0.58*** | -0.00 | -0.42*** | -0.46*** | -0.31** |
| Good | — | 0.83*** | 0.85*** | 0.52*** | 0.67*** | 0.71*** | 0.27* |
| Safe |  | — | 0.50*** | 0.33** | 0.47*** | 0.62*** | 0.30* |
| Enticing |  |  | — | 0.23 | 0.62*** | 0.57*** | 0.10 |
| Alive |  |  |  | — | 0.45*** | 0.44*** | 0.33** |
| Improvable |  |  |  |  | — | 0.59*** | 0.33** |
| Regenerative |  |  |  |  |  | — | 0.38*** |
| Just |  |  |  |  |  |  | — |

**Table S6**

*Correlations among Cystic Fibrosis Patients (Study 1)*

|  | Good | Safe | Enticing | Alive | Improvable | Regenerative | Just |
| --- | --- | --- | --- | --- | --- | --- | --- |
| Emotional Distress | -0.58*** | -0.59*** | -0.46*** | -0.28** | -0.53*** | -0.56*** | -0.54*** |
| Good | — | 0.87*** | 0.90*** | 0.61*** | 0.72*** | 0.73*** | 0.58*** |
| Safe |  | — | 0.63*** | 0.44*** | 0.61*** | 0.66*** | 0.56*** |
| Enticing |  |  | — | 0.41*** | 0.66*** | 0.60*** | 0.39*** |
| Alive |  |  |  | — | 0.53*** | 0.55*** | 0.63*** |
| Improvable |  |  |  |  | — | 0.68*** | 0.61*** |
| Regenerative |  |  |  |  |  | — | 0.61*** |
| Just |  |  |  |  |  |  | — |

**Table S7**

*Study 1 Regressions with Focal Beliefs and their Interactions Terms Predicting Emotional Distress*

| Model | 1. Regenerative | 2. Improvable | 3. Just |
| --- | --- | --- | --- |
| Current Illness Dummy | 1.27[0.76, 1.77]*** | 1.41[0.90, 1.92]*** | .98[0.60, 1.36]*** |
| World Belief | -.26[-.35, -.18]*** | -.17[-.26, -.08]*** | -.14[-.22, -.06]*** |
| Illness * World Belief | -.34[-.49, -.18]*** | -.35[-.50, -.20]*** | -.31[-.45, -.16]*** |
| Age | -.01[-.02, -.01]*** | -.01[-.02, -.01]*** | -.01[-.02, -.01]*** |
| Sex (F=1, M=0) | .08[-.05, .21]^ns^ | .03[-.10, .16]^ns^ | .01[-.13, .14]^ns^ |

Note: Values show unstandardized coefficients and 95% confidence intervals. ***p<.001, ^ns^=nonsignificant.

**Additional Results for Study 2**

**Figure S1**

*Correlations between Secondary Primal World Beliefs and Stress in Waves 1-3 (Study 2)*

**
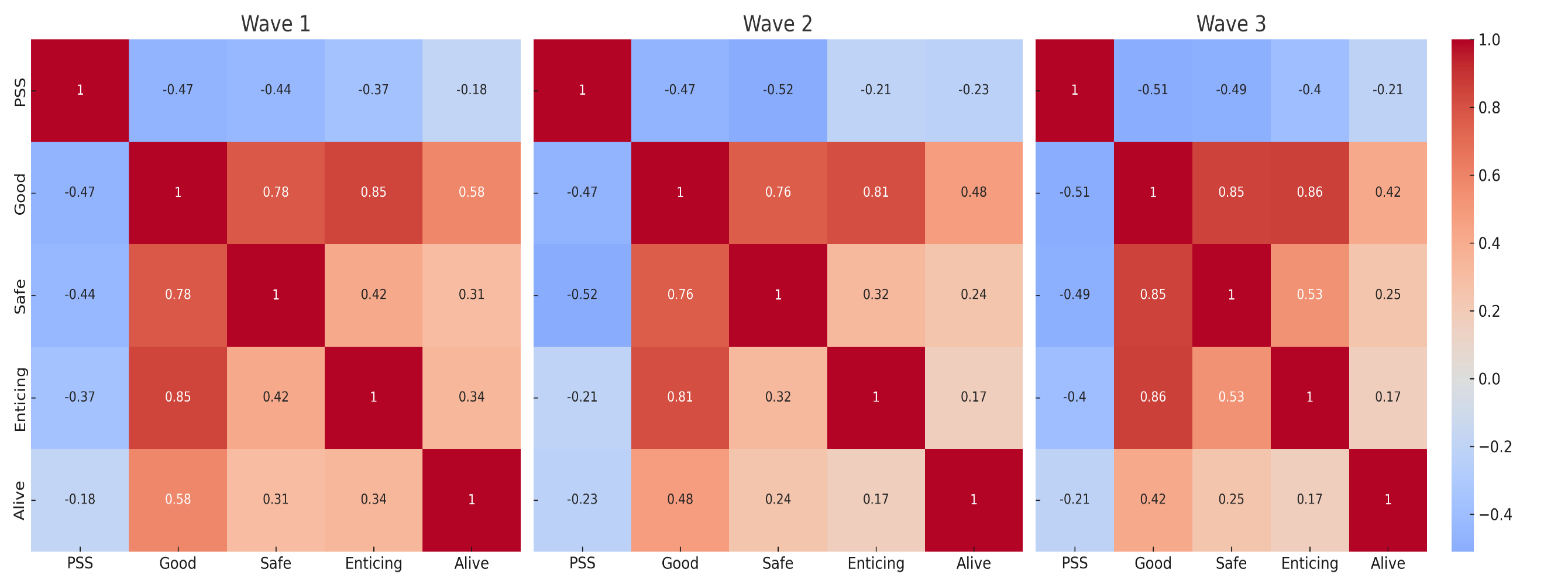
**

**Moderated Regression Models in Study 2**

**Table S8**

*Regression Model for Good*

|  | **PSS_ave** | | |
| --- | --- | --- | --- |
| *Predictors* | *Estimates* | *CI* | *p* |
| (Intercept) | 3.07 | 2.98 – 3.17 | **<0.001** |
| Good trait | -0.47 | -0.64 – -0.31 | **<0.001** |
| afterShooting | 0.16 | 0.05 – 0.28 | **0.004** |
| Good X After Shooting | -0.20 | -0.41 – 0.01 | 0.057 |
| **Random Effects** | | | |
| σ^2^ | 0.20 | | |
| τ_00_ _userID_ | 0.18 | | |
| ICC | 0.48 | | |
| N _userID_ | 116 | | |
| Observations | 286 | | |
| Marginal R^2^ / Conditional R^2^ | 0.184 / 0.574 | | |

**Table S9**

*Regression Model for Safe Only (Study 2)*

|  | **PSS_ave** | | |
| --- | --- | --- | --- |
| *Predictors* | *Estimates* | *CI* | *p* |
| (Intercept) | 3.08 | 2.99 – 3.18 | **<0.001** |
| Safe trait | -0.43 | -0.58 – -0.27 | **<0.001** |
| After Shooting | 0.18 | 0.07 – 0.29 | **0.002** |
| Safe X After Shooting | -0.27 | -0.46 – -0.08 | **0.005** |
| **Random Effects** | | | |
| σ^2^ | 0.20 | | |
| τ_00_ _userID_ | 0.18 | | |
| ICC | 0.48 | | |
| N _userID_ | 116 | | |
| Observations | 286 | | |
| Marginal R^2^ / Conditional R^2^ | 0.196 / 0.583 | | |

**Table S10**

*Regression Model for Enticing Only (Study 2)*

|  | **Stress (PSS)** | | |
| --- | --- | --- | --- |
| *Predictors* | *Estimates* | *CI* | *p* |
| (Intercept) | 3.07 | 2.97 – 3.17 | **<0.001** |
| Enticing trait | -0.38 | -0.53 – -0.23 | **<0.001** |
| After Shooting | 0.17 | 0.06 – 0.29 | **0.004** |
| Enticing X After Shooting | -0.04 | -0.23 – 0.14 | 0.639 |
| **Random Effects** | | | |
| σ^2^ | 0.21 | | |
| τ_00_ _userID_ | 0.20 | | |
| ICC | 0.50 | | |
| N _userID_ | 116 | | |
| Observations | 286 | | |
| Marginal R^2^ / Conditional R^2^ | 0.138 / 0.566 | | |

**Table S11**

*Regression Model for Alive Only (Study 2)*

|  | **Stress (PSS)** | | |
| --- | --- | --- | --- |
| *Predictors* | *Estimates* | *CI* | *p* |
| (Intercept) | 3.06 | 2.95 – 3.17 | **<0.001** |
| Alive trait | -0.10 | -0.20 – 0.00 | 0.058 |
| After Shooting | 0.16 | 0.04 – 0.27 | **0.007** |
| Alive X After Shooting | -0.11 | -0.23 – -0.00 | **0.042** |
| **Random Effects** | | | |
| σ^2^ | 0.20 | | |
| τ_00_ _userID_ | 0.25 | | |
| ICC | 0.55 | | |
| N _userID_ | 116 | | |
| Observations | 286 | | |
| Marginal R^2^ / Conditional R^2^ | 0.051 / 0.577 | | |
